# Simplified Process Model Discovery Based on Role-Oriented Genetic Mining

**DOI:** 10.1155/2014/298592

**Published:** 2014-01-29

**Authors:** Weidong Zhao, Xi Liu, Weihui Dai

**Affiliations:** ^1^Software School, Fudan University, No. 220 Handan Road, Shanghai 200433, China; ^2^School of Management, Fudan University, No. 220 Handan Road, Shanghai 200433, China

## Abstract

Process mining is automated acquisition of process models from event logs. Although many process mining techniques have been developed, most of them are based on control flow. Meanwhile, the existing role-oriented process mining methods focus on correctness and integrity of roles while ignoring role complexity of the process model, which directly impacts understandability and quality of the model. To address these problems, we propose a genetic programming approach to mine the simplified process model. Using a new metric of process complexity in terms of roles as the fitness function, we can find simpler process models. The new role complexity metric of process models is designed from role cohesion and coupling, and applied to discover roles in process models. Moreover, the higher fitness derived from role complexity metric also provides a guideline for redesigning process models. Finally, we conduct case study and experiments to show that the proposed method is more effective for streamlining the process by comparing with related studies.

## 1. Introduction

Information systems are mostly driven by the process model. Therefore, the process model is a key factor for the system to run effectively. Process mining techniques aim to automatically generate process models by analyzing the event log, which assist in the redesign of process models.

Process mining first appeared in the field of software engineering. It is proposed by Jonathan from New Mexico State University in 1995 [1]. Then, Agrawal started to apply process mining to process management in 1998 [2]. He used directed graphs to represent the association between different activities in business processes. Instead of using directed graph, Aalst used the workflow net, which is a subclass of Petri nets to represent process models. Based on the work, some scholars extended process mining algorithm to handle business logic, including sequence, parallel and circular relationship [3]. Compared with other process mining algorithms, genetic mining proposed by de Medeiros et al. is a global search algorithm, dealing with noise effectively [4].

The structure of process models is often complex. They consist of circular, parallel, choice and hidden structures. Current process mining algorithms are not well developed in dealing with these structures. Process mining aims to find the mode from the process execution log which most closely matches the actual behavior of business processes. But with the complication of the process, there will be large amounts of alternative process models. How to find out the process model with low complexity is necessary for process improvement. For example, Tian proposed a process mining algorithm combining genetic algorithms and simulated annealing algorithm [5]. Through analyzing process participants, the algorithm built a causal relationship matrix mapping process instances to the population chromosomes and mined the process model effectively. In fact, those methods are very complex, and the process model mined may be very complicated too. Then, some researchers employed the complexity metric of control flow and computed the structural complexity of process models to guide process redesign [6]. However, these approaches only pay attention to the complexity of the process model from the perspective of control flow. How to discover process models with low complexity, especially from the organizational view, and streamline the collaboration between process actors are necessary.

At present, most of process mining methods are based on process activities. They neglect the fact that the process depends on the collaboration between multiple roles. Though some scholars come to extract knowledge from the role perspective, their studies on the relationship between process roles are not complete and merely confined to discuss the interaction among organizational entities [7, 8]. Actually, the relationship between them is very complicated, so it is hard to uncover hidden information and the role complexity of business processes is ignored.

The remainder of the paper is organized as follows.  Section 2 introduces the role complex metrics. Then, the role complex fitness is shown in Section 3 with the case study conducted in Section 4. Moreover, we conduct comparative experiments in Section 5. Finally, Section 6 concludes the paper.

## 2. The Role Complexity of Process Models

The complexity of business processes describes process models from different perspective. It implies whether a business process model has right size, clear structure and is easy to understand and reasonably modular. Therefore, it is necessary to design process models with low complexity.

The previous studies focus on control flow that is composed of activities and their relationships. For example, Cardoso discussed the complexity metric of control flow through experiments [9]. In addition, Vanderfeesten et al. proposed cohesion and coupling metrics for process design [10]. However, a process is the integration of participants (roles), resources, objectives, information and business rules, and so on. Control flow is just one of the factors affecting process complexity. More researchers begin to analyze business processes from different aspects.

### 2.1. The Role Cohesion Metric

The role cohesion analyzes closeness of multiple activities performed by one role. It proposes that the activities performed by the same role have closer relationship. For example, if the activities performed by a role are based on the same data or require similar capacities, then the role may have greater role cohesion and be more efficient to take the activities. The role cohesion metric is categorized into the following types.

(1) The role activity cohesion is to assess the interaction between roles in terms of control flow. The shorter the interval is, the higher the role activity cohesion is. Herein, the interval between two activities is defined as the number of activities between them. For example, there are *n* activities between the activities *a*_1_and *a*_2_. Then, we can define the distance between them as *L*(*a*_1_, *a*_2_) = *n* + 1. Actually, there may be several execution sequences containing *a*_1_ and *a*_2_. Say that there are *v* execution sequences which contain *a*_1_ and *a*_2_, we can define the distance between *a*_1_ and *a*_2_ as

(1) $$\begin{array}{c} L\left(a_{1},a_{2}\right)=\overset{v}{\underset{k\;=\;1}{\sum }}\frac{L_{k}\left(a_{1},a_{2}\right)}{v}. \end{array}$$

We can examine the interval of every two activities by a role to measure the role activity cohesion. So, the role activity cohesion of *r* can be defined as

(2) $$\begin{array}{c} \alpha_{r}=\frac{\left(1+\sum_{a_{1}\neq a_{2};a_{1},a_{2}\in T(r)}^{}\left(\max L-L\left(a_{1},a_{2}\right)\right)/2\right)}{\left(C_{T\left(r\right)}^{2}\times \max L\right)}, \end{array}$$

where *T*(*r*) represents all activities performed by *r*, max *L* is the maximum distance between activities of the process separately, and *C*_*T*(*r*)_^2^ means the number of situations of activities' combinations. So, the role activity cohesion reflects the distance of activities performed by *r*. The shorter the distance is, the higher the role activity cohesion of *r* is.

(2) The role data cohesion measures the cohesion between roles in terms of data. It analyzes the frequency of using different data.

Provided that *a is the input data set of *a*, which is necessary for *a*, and a* is the output data set of *a*, then *I* = *a ∪  a* is the data set, which is related to *a*, and |*I*| is the number of elements in *I*. Then, the role data cohesion of *r* is defined as

(3) $$\begin{array}{c} \beta_{r}=\frac{\left(1+\sum_{a_{1}\neq a_{2};a_{1},a_{2}\in T(r)}^{}\left|I_{1}\cap I_{2}\right|/2\right)}{\left(\sum_{a_{1}\neq a_{2};a_{1},a_{2}\in T(r)}^{}\left|I_{1}\cup I_{2}\right|/2\right)}. \end{array}$$

The role data cohesion indicates the proportion the input and output data of activities by *r* have in common. The more they share the same data, the higher the role data cohesion of *r* is.

(3) The role ability cohesion measures the cohesion between roles in terms of abilities needed. It computes what abilities are required for the role to perform different activities. If *E* is the set of abilities necessary to perform *a*, and |*E*| is the number of elements in *E*, then the role ability cohesion of *r* is defined as

(4) $$\begin{array}{c} \chi_{r}=\frac{\left(1+\sum_{a_{1}\neq a_{2};a_{1},a_{2}\in T(r)}^{}|E_{1}\cap E_{2}|/2\right)}{\left(\sum_{a_{1}\neq a_{2};a_{1},a_{2}\in T(r)}^{}|E_{1}\cup E_{2}|/2\right)}. \end{array}$$

The role ability cohesion shows the kinds of common abilities required by different activities performed by *r*. The more they share, the higher the role ability cohesion of *r* is.

As a whole, the role cohesion metric is computed as follow:

(5) $$\begin{array}{c} \text{Co}{\text{h}}_{r}=\alpha_{r}\times \beta_{r}\times \chi_{r}. \end{array}$$

### 2.2. The Role Coupling Metric

The role coupling metric implies the degree of association between activities taken by different roles. If there are several kinds of connections between activities performed by two roles, and one role is connected with more roles, it has greater role coupling metric.

(1) The role activity coupling shows the degree of association between activities performed by different roles in a process. If activities by different roles are connected, these roles are interrelated. There are several kinds of connections corresponding to different degrees of association.

Assume that *r* is responsible for *a*_1_ and *a*_2_ is not performed by role *r*. *m* and *n* represent the outdegree and indegree of the connector between *a*_1_ and *a*_2_ separately. We can define the coupling weight as follows through the connection form between *a*_1_ and *a*_2_.

- If *a*_1_ and *a*_2_ are directly connected, then *a*_1_ and *a*_2_ are coupled, so the coupling weight between them is 1.
- If *a*_1_ and *a*_2_ are connected through AND connector, then *a*_1_ and *a*_2_ are also coupled, so the coupling weight between them is 1.
- If *a*_1_ and *a*_2_ are connected through OR connector, then the probability of coupling between them is (2^*m*−1^ × 2^*n*−1^)/((2^*m*^ − 1) × (2^*n*^ − 1)), so the coupling weight between them is (2^*m*−1^ × 2^*n*−1^)/((2^*m*^ − 1) × (2^*n*^ − 1)).
- If *a*_1_ and *a*_2_ are connected through XOR connector, then the probability of coupling and coupling weight between them are both 1/mn.
- If *a*_1_ and *a*_2_ are not connected, they cannot be coupled, so the coupling weight between them is 0.

The role coupling metric of *r* is defined as

(6) δr =∑a1∈T(r);a2∉T(r)[connected(a1,a2)+connected(a2,a1)]|Arc|,

where connected (*a*_1_, *a*_2_) represents the coupling weight between *a*_1_ and *a*_2_, Arc stands for the set of arcs in the process model, and |Arc| is the number of elements in Arc. The larger *δ*_*r*_ is, the higher the role activity coupling of *r* is.

(2) The role coupling is not only related to role activity coupling, but also to the number of roles associated with a role. If a role is associated with more roles, it may be complicated. So, the role relation coupling of *r* is defined as

(7) $$\begin{array}{c} \varepsilon_{r}=\frac{N_{r}}{\left|R\right|}, \end{array}$$

where *N*_*r*_ is the number of roles associated with *r* and |*R*| represents the number of roles in the process. The larger the *ε*_*r*_ is, the higher the role relation coupling of *r* is. The role coupling metric is defined as

(8) $$\begin{array}{c} \text{Co}{\text{u}}_{r}=\delta_{r}\times \varepsilon_{r}. \end{array}$$

The lower the role cohesion is and the higher the role coupling is, the more complex the role is. Therefore, the role complexity is defined as

(9) $$\begin{array}{c} C_{r}=\frac{\text{Co}{\text{u}}_{r}}{\text{Co}{\text{h}}_{r}}. \end{array}$$

As each role is different in importance, the role complexity of each role should be accompanied by the appropriate weight depending on its importance. Then, according to the weight of each role and its role complexity, we can get the role complexity of a business process. The weight of each role can be defined as

(10) $$\begin{array}{c} W_{r}=\frac{1}{3}\times \left(\frac{t}{T}+\frac{\text{time}}{\text{TIME}}+\frac{\text{cost}}{\text{COST}}\right), \end{array}$$

where *t*, time, and cost represent the number of activities, time, and cost to perform activities by *r*separately. *T*, TIME, and COST represent the number of activities, time, and cost to perform activities of the business process separately. In (10), 1/3 is to ensure that the sum of weights of all the roles is 1. The role complexity of the business process is defined as

(11) $$\begin{array}{c} C=\sum_{r\in R}W_{r}\times C_{r}, \end{array}$$

where *C*_*r*_ is the role complexity of the role *r*, *W*_*r*_ represents its weight, and *R* is the set of roles in the process.

## 3. The Fitness Function of Role-Oriented Process Mining

In 2005, Aalst first introduced genetic algorithm to process mining (genetic mining). In genetic mining, an individual is a candidate process model and the fitness function evaluates how well it is able to represent the actual process [6].

The fitness function is used to evaluate the adaptation of every individual and guide searching process of genetic programming. In order to mine the simplified business process model, we introduce the complex fitness into the fitness function.

### 3.1. Role Complexity Fitness

We define *C*(*R*) as role complexity of process individual; min⁡(*C*) and max⁡(*C*) stand for the minimum role complexity value and the maximum role complexity value separately in a generation of population. So, the role complexity fitness is defined as

(12) $$\begin{array}{c} \text{P}{\text{F}}_{\text{Complex}}\left(R\right)=\frac{C\left(R\right)-\min\left(C\right)}{\max\left(C\right)-\min\left(C\right)}. \end{array}$$

PF_complex_ describes the relative role complexity of individuals in the same population in (12). When the role complexity value of an individual is the maximum, the fitness value of role complexity is 1. When the role complexity value of individual reaches the minimum, the fitness value of role complexity is 0. The smaller the PF_complex_ of the individual is, the lower its relative complexity is.

### 3.2. Fitness Function

The basic principle of fitness function is that a process model should match event logs as much as possible. So the precision is defined as

(13) $$\begin{array}{c} \text{P}{\text{F}}_{\text{precise}}\left(R\right)=\overset{\left|R\right|}{\underset{i=1}{\sum }}\overset{}{\underset{p_{1}!=p_{2}\&p_{1},p_{2}\in f(R_{i})}{\sum }}\frac{\cos\left(p_{1},p_{2}\right)}{2}, \end{array}$$

where |*R*| means the number of roles in a process model, *f*(*R*_*i*_) is the participant set of *R*_*i*_, and cos⁡(*p*_1_, *p*_2_) is the cosine similarity between the participants *p*_1_ and *p*_2_.

In order to discover simple process models, we add the role complexity fitness to describe the precision. As mining process models with correct roles is the nature of process mining, complex fitness should have lower weights. The individuals that are complex are punished. Assuming that the weight of the precision and complexity fitness are *λ* and *θ* separately, the complete fitness is defined as follows:

(14) $$\begin{array}{c} \text{PF}\left(R\right)=\lambda {\text{PF}}_{\text{precise}}\left(R\right)-\theta {\text{PF}}_{\text{complex}}\left(R\right), \end{array}$$

where the fitness is affected by not only the correct recognition of roles in the process model, but also by the role complexity of the model. So, it can make the role complexity of mined process models lower.

The basic idea of genetic mining is as follows. First, event logs are collected and activities by each participant are analyzed. Then, initial population is created. After that, the fitness of every individual in the population is computed according to the fitness function (14). If the fitness does not satisfy the termination condition, the population needs iterative evolution through the genetic operations including selection, crossover, and mutation. Each genetic operation transfers the individual, which has higher fitness value in the population to the next generation. This loop terminates until the optimal solution is found. In Section 4, we resort to a case study to discuss the procedure of genetic mining in detail.

## 4. Case Study

We first give a process mining experiment mentioned in [11] and compare its process mining algorithm with ours.

The interaction between roles and role identification are analyzed by using genetic algorithm and achieving optimal role identification [11]. On the one hand, it shows the degree of similarity in the activities executed by participants. On the other hand, it indicates the similarity of performing internal activities of the participants of a certain role and the interaction between participants.  Table 1 shows the fragment of workflow logs. The data in the first column represents the process instance number, the second column represents activities, and the third column represents the participant corresponding to the activity in the second column.

The matrix *M* shows the role situation of a process model *Q*. If *M*_*jk*_ is 1, that means the participants *j* and *k* undertake the same role. If *M*_*jk*_ is 1 and *j* = *k*, that means*j* undertakes the role by himself [11]. As seen in matrix *M*, *p*101, *p*105, and *p*115 undertake a role, *p*106 undertakes a role alone, and *p*107 and *p*114 undertake a role. We can encode the process model *Q* through linking value of each row in *M*, and the chromosome is 010001000011000010000

(15) M=p101p105p106p107p114p115(p101p105p106p107p114p115010001000011000010000).

In workflow logs, the situation of activities by each actor is as follows: *p*101 executes *a* 10 times, *b* 6 times, and *i* 5 times separately. *p*105 executes *c* 2 times, *d* 2 times, *e* 5 times, and *f* 6 times separately. *p*106 executes *h* 8 times. *p*107 executes activity *a* 8 times, *b* 7 times, and *i* 6 times separately. *p*114 executes activity *c* 7 times, *d* 7 times, *e* 8 times, *f* 6 times, *g* 10 times, and *h* 2 times separately. *p*115 executes *b* 5 times, *c* 3 times, *d* 3 times, *e* 5 times, *f* 4 times, and *h* 1 times separately. Tables 2 and 3 show the data and abilities required for each activity in the process separately.

The precision value and the role complexity of *Q* represented in the matrix *M* are as follows:

(16) PFPrecise(R)=∑i=1|R|∑p1!=p2;p1,p2∈f(Ri)cos⁡(p1,p2)2|f(Ri)|≈1.33CR=CR1+CR2+CR3≈392.19.

The maximum role complexity value in this generation is 393.74, and the minimum one is 25.54. So, the complex fitness value of *Q* is

(17) $$\begin{array}{c} \text{P}{\text{F}}_{\text{Complex}}\left(R\right)=\frac{C\left(R\right)-\min\left(C\right)}{\max\left(C\right)-\min\left(C\right)}\approx 0.996. \end{array}$$

It is supposed that the complex fitness has lower weight than the precise fitness. In this paper, we assume that the weight of the precision fitness is 0.7 and that of the complex fitness is 0.3. So, the fitness value of *Q* is

(18) $$\begin{array}{c} \text{PF}\left(R\right)=\lambda {\text{PF}}_{\text{Precise}}\left(R\right)-\theta {\text{PF}}_{\text{Complex}}\left(R\right)=0.632. \end{array}$$

After that, genetic operations are performed: we use the selection operator. It retains process models which have higher fitness values. Herein, we choose 15 process models to get corresponding chromosomes in each generation. Then, we use the crossover operator. For example, the model *A* with the code 010000000001000010001 makes a change in the 16th bit with the model *B* with the code 010001000011000110000, the new chromosomes 010000000001000110001 and 010001000011000010000 are produced. Its occurrence probability is *P*_*c*_:

(19) $$\begin{array}{c} P_{c}=\frac{C_{1}\left(f_{\max}-f_{m}\right)}{f_{\max}-f_{\min}}+C_{2}\approx 0.58, \end{array}$$

where *C*_1_ and *C*_2_ are constants, and we assume that they are 0.7 and 0.1. *f*_max⁡_ and *f*_min⁡_ are the maximum and minimum fitness values in this generation separately: 0.87 and 0.52. *f*_*m*_ is the fitness value of *Q*, which is 0.632.

The mutation operation is that one bit of the chromosome changes at random, from 0 to 1 or from 1 to 0. Its probability of occurrence is 1 − (1 − *P*_*m*_)5 and *P*_*m*_ is defined as

(20) $$\begin{array}{c} P_{m}=\frac{C_{3}\left(f_{\max}-f_{m}\right)}{f_{\max}-f_{\min}}+C_{4}\approx 0.07, \end{array}$$

where *C*_3_ and *C*_4_ are constants and we assume that they are 0.09 and 0.01 separately.

As mentioned above, we can get the role situation matrix *MA* through role-oriented genetic mining below:

(21) MA=p101p105p106p107p114p115(p101p105p106p107p114p115000100000111000000110).

**Figure 1:**
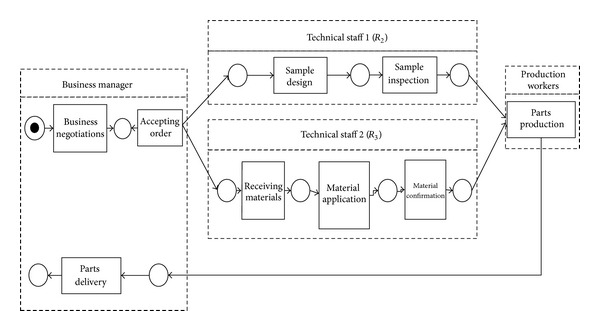
The mined role-activity diagram.

As seen from *MA*,   *p*101 and *p*107 share a role,*p*105,   *p*114, and *p*115 undertake a role, and both *p*106 and *p*114 undertake a role alone. It groups the participants into four roles: business manager, technical staff (*R*_2_), technical staff (*R*_3_), and production workers.  Figure 1 is the role-activity diagram through our role-oriented process mining method.

In order to verify the effectiveness of our method, we compare the role complexity of process models mined by our algorithm and the algorithm proposed in [11]. For Figure 1, we can calculate the role complexity of *R*_2_ and *R*_3_ by our algorithm. The cohesion and coupling complexity of *R*_2_ and *R*_3_ are shown in Table 4.

*R*_2_ and *R*_3_ are taken as one role [11]. The role complexity of that role is 7.82. Clearly, the role complexity of that role is far greater than the sum of the role complexity of *R*_2_ and *R*_3_ in Figure 1. The reason is that the technical staff is mainly responsible for designing samples and inspection, as well as raw material application and confirmation. These two kinds of activities are different and require different data and abilities, which leads to low role activity cohesion, role data cohesion, and role ability cohesion. This brings about the high role complexity ultimately. By means of our method, the work of technical staff is split, which ensures that the process model is correct and has low role complexity at the same time. The result shows that our method performs better in discovering simplified role-based process models. In fact, the roles have high cohesion and low coupling.

The role mining algorithms proposed in [12, 13] got role hierarchy through the combination of permissions based on participants and their permissions. Their algorithm identified roles based on permissions, ignorant of the difference between the activities of participants. Phalp and Shepperd measured the role's complexity through surveying internal activities and interactions between roles [14]. They didnot give full considerations of cohesion and coupling between roles. In addition, [15] considered the complexity of the application of resources. But it ignored the internal cohesion of roles. In comparison, our method treats the similarity between activities by different participants as the basis for identifying roles and it is based on genetic mining. So, it deals with noise more effectively in workflow logs. Additionally, it measures the complexity of roles through cohesion and coupling in terms of activities and resources. Therefore, the role complexity makes the process model correct and simple.

## 5. Experiments

In order to analyze the performance of the algorithm we proposed, we select some event logs produced by 8 workflow models shown partly in Table 1 to perform some experiments.

**Figure 2:**
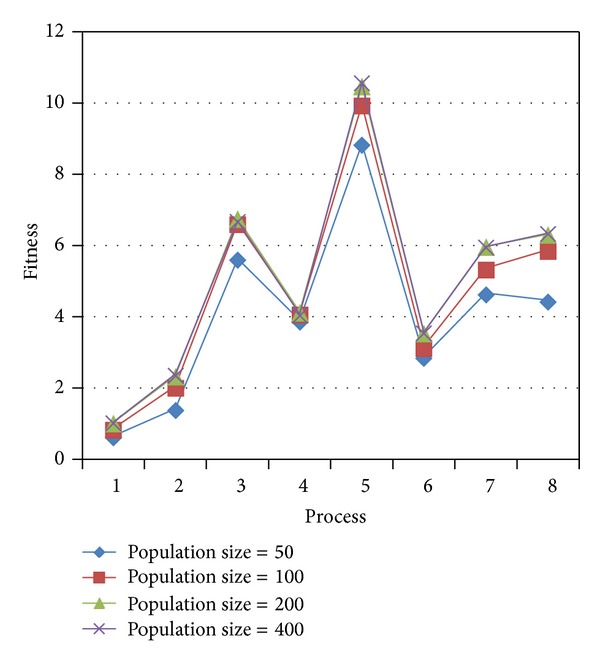
Comparison of different population sizes.

As can be seen from Figure 2, when the population size is small, the fitness value is low. And when the population size is bigger than 200, the fitness value no longer increases. So, the population size is set to 200.

**Figure 3:**
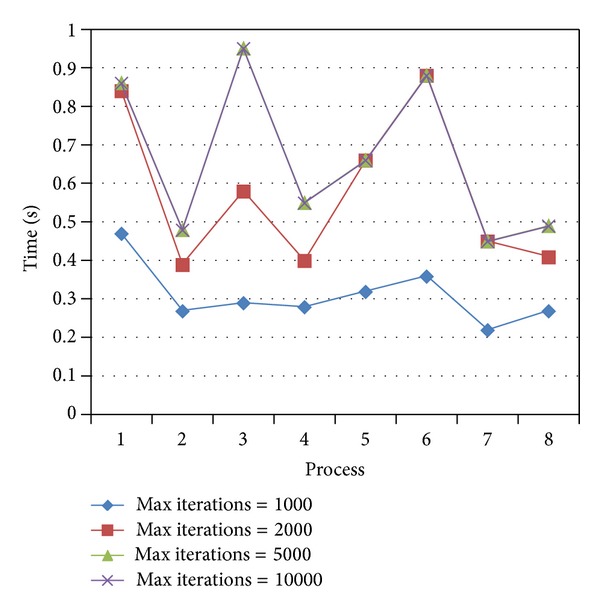
Comparison of different maximum iterations.

As shown in Figure 3, the time spent by the algorithm is increased with the increase of the maximum number of iterations. When the maximum number of iterations is small, the algorithm will stop before finding the optimal solution. In this case, the solution is questionable. And when the maximum number of iterations reaches 5000, the time spent by the algorithm will remain stable. That means the optimal solution will be found before 5000 iterations. So, the maximum number of iterations is set to 5000.

**Figure 4:**
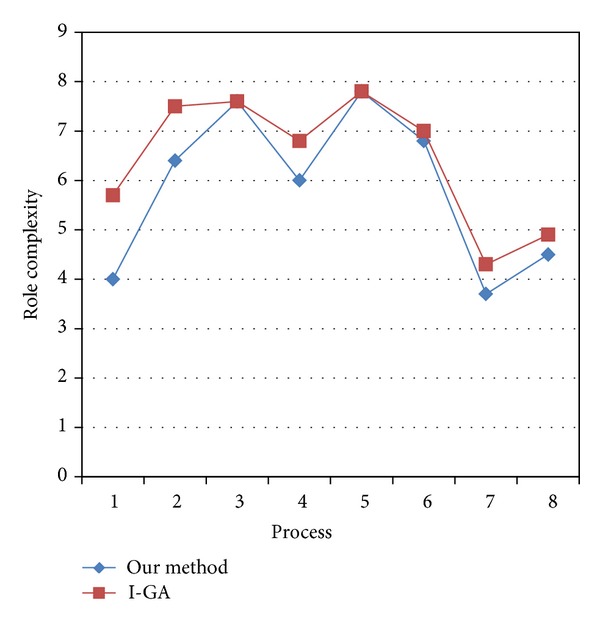
Comparison of role complexity.

Except for processes 3 and 5, the role complexity of process models mined by our method is lower than that by I-GA [4] in Figure 4. The reason is that the role complexity of process models is not considered in I-GA [4]. And in processes 3 and 5, the role complexity of process models may be not reduced any more. Therefore, the algorithm we propose can reduce the role complexity of mined process models.

**Figure 5:**
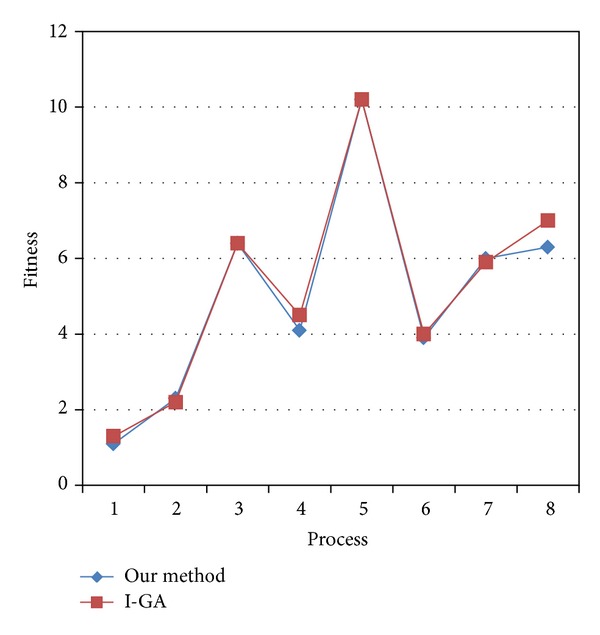
Comparison of fitness.

In Figure 5, we can see that the fitness value of process models mined by our method is relatively close. That means, though our method considers role complexity, it has little adverse effect on the fitness.

Through these experiments, we can see that the algorithm performs better when mining simpler process models, because it uses the role complexity of process models. Therefore, it can reduce the role complexity when mining process models.

## 6. Conclusions

In this paper, we combine genetic programming with the role complexity and propose the role-oriented process mining approach. The advantage of our method is that it can mine process models not only correctly, but also simply. In the future, we will consider the relationship between process roles more comprehensively and reduce the role complexity further. Besides, we can improve the efficiency of model mining through improved genetic algorithm.

## Figures and Tables

**Table 1:** The fragment of workflow log.

Instance number	Activities	Participants
115	Business negotiations (a)	*p*101
115	Accepting order (b)	*p*101
116	Business negotiations (a)	*p*107
115	Sample design (c)	*p*114
115	Sample inspection (d)	*p*114
116	Accepting order (b)	*p*107
117	Business negotiations (a)	*p*101
115	Receiving materials (e)	*p*105
116	Receiving materials (e)	*p*105
115	Material application (f)	*p*105
115	Material confirmation (g)	*p*105
115	Parts production (h)	*p*106
115	Parts delivery (i)	*p*101
117	Business negotiations (a)	*p*101

**Table 2:** Data required for each activity.

Activity	Data elements
Sample design	Order contracts, sample instructions, sample
Sample inspection	Sample instructions, sample, sample inspection form
Receiving materials	Order contracts, materials receipt form
Material application	Material application form, materials receipt form, materials confirmation form, materials
Material confirmation	Material application form, materials receipt form, materials confirmation form, materials
Parts production	Materials, sample, materials confirmation form, sample inspection form, finished parts
Parts delivery	Finished parts

**Table 3:** Abilities required for each activity.

Activity	Required ability
Sample design	Sample design ability, sample identification ability
Sample inspection	Sample identification ability
Receiving materials	Material identification ability
Material application	Material identification ability
Material confirmation	Material identification ability
Parts production	Production ability
Parts delivery	Delivery ability, negotiation ability

**Table 4:** Cohesion and coupling complexity.

	*R*_2_	*R*_3_
Role activity cohesion	1	5/6
Role data cohesion	3/4	1/2
Role ability cohesion	1	4/3
Role activity coupling	2/9	2/9
Role relation coupling	1/2	1/2
Role complexity	0.15	0.20
